# Macrophages in homeostatic immune function

**DOI:** 10.3389/fphys.2014.00146

**Published:** 2014-05-05

**Authors:** Jonathan Jantsch, Katrina J. Binger, Dominik N. Müller, Jens Titze

**Affiliations:** ^1^Mikrobiologisches Institut – Klinische Mikrobiologie, Immunologie und Hygiene, Universitätsklinikum Erlangen und Friedrich-Alexander-Universität Erlangen-NürnbergErlangen, Germany; ^2^Experimental and Clinical Research Center (ECRC), Max-Delbrück Center for Molecular Medicine, Charité Medical FacultyBerlin, Germany; ^3^Interdisciplinary Center for Clinical Research and Department of Nephrology and Hypertension, Friedrich-Alexander-Universität Erlangen-NürnbergErlangen, Germany; ^4^Divison of Clinical Pharmacology, Vanderbilt University School of MedicineNashville, TN, USA

**Keywords:** macrophage polarization, arteriogenesis, angiogenesis, lipid metabolism, peroxisome proliferator activator receptor (PPAR), salt, Na^+^ storage, tonicity enhance binding protein (TonEBP)/nuclear factor of activated T-cells 5(NFAT5)

## Abstract

Macrophages are not only involved in inflammatory and anti-infective processes, but also play an important role in maintaining tissue homeostasis. In this review, we summarize recent evidence investigating the role of macrophages in controlling angiogenesis, metabolism as well as salt and water balance. Particularly, we summarize the importance of macrophage tonicity enhancer binding protein (TonEBP, also termed nuclear factor of activated T-cells 5 [NFAT5]) expression in the regulation of salt and water homeostasis. Further understanding of homeostatic macrophage function may lead to new therapeutic approaches to treat ischemia, hypertension and metabolic disorders.

## Introduction

Leukocytes, consisting of diverse cell types, are the cellular constituents of the body's immune system. As they patrol throughout the blood and lymphatic systems, these cells become recruited to infected or damaged tissue and then act to restore the integrity of the site. Leucocytes share a common origin from haematopoietic stem cells and develop into a variety of subsets according to distinct differentiation stimuli. The mononuclear phagocyte system (MPS) constitutes a subgroup of leukocytes comprising monocytes, macrophages, and dendritic cells; the latter representing specialized antigen-presenting cells linking innate and adaptive immune responses (reviewed in Geissmann et al., 2010).

The cells of the MPS are not only involved in inflammatory and anti-infective processes but also play an important homeostatic role in maintaining the steady state of the tissue (Medzhitov, 2008; Lutz and Kurts, 2009; Pollard, 2009); for example, monocytes derived from the spleen act to rapidly promote tissue repair after myocardial infarction (Swirski et al., 2009). Particularly, macrophages have essential and diverse roles in regulating tissue homeostasis. Over a century ago Metchnikoff proposed that macrophages are not only involved in combating invading intruders, but play other roles within the body to maintain homeostasis. He used the terms “physiological” and “pathological” (e.g., induced by pathogens) tissue insults to describe the initial “disharmony” facing different cells within a multicellular organism. Whilst these two types of insults have different and competing demands on the tissue, in both situations macrophages have a central homeostatic role to re-establish the steady state of the tissue (Tauber, 2003). Recent classification proposes that macrophages have a continuum of phenotypic subsets, each with overlapping functions ranging from classically activated (M1) to alternatively activated (M2) macrophages, where the latter includes both wound healing and regulatory macrophages (Gordon, 2003; Mosser and Edwards, 2008; Murray and Wynn, 2011; Locati et al., 2013; Mantovani et al., 2013). In addition to these well recognized activities, macrophages are important for various other functions including tissue development, such as neuronal patterning, bone morphogenesis, and generation of adipose tissue (Pollard, 2009), promoting angiogenesis and arteriogenesis (Murdoch et al., 2008; Pollard, 2009), and the maintenance of internal body fluids or the “milieu interieur.” Regulation of the *mileu interiur* is especially important for tissue homeostasis as, according to Bernard, this guarantees a “free and independent” life (Bernard, 1957). In this review, we will summarize recent developments of the role of macrophages in regulating blood supply and metabolism. Finally, we will highlight the role of macrophages as central regulators of internal body fluids *via* the expression of NFAT5. Further understanding into this emerging concept of homeostatic macrophage function may offer new insight into the regulation of energy and electrolyte metabolism, and thus offer new therapeutics to so-called “Western diseases.”

## Macrophages as angiogenic and arteriogenic accessory cells

Tissue hypoxia drives the development of new blood vessels from existing blood vessels (angiogenesis), and the remodeling of existing collateral vessels (arteriogenesis), in order to ensure sufficient tissue perfusion and hence, tissue oxygenation (Potente et al., 2011). It has been shown that macrophages play an important role in both processes, as angiogenic, and arteriogenic accessory cells (Pollard, 2004; Murdoch et al., 2008; Coffelt et al., 2009; David Dong et al., 2009; Nucera et al., 2011; Chambers et al., 2013; Owen and Mohamadzadeh, 2013). Myeloid cells are first attracted to the site of injury, for example by the chemokine CCL2 (Low-Marchelli et al., 2013). Accordingly, interference with local monocyte attraction to ischemic tissue resulted in flap necrosis due to impaired flap revascularization (Khan et al., 2013). Once at the ischemic site, macrophages are exposed to vessel- and tissue-derived cytokines [such as Angiopoietin (ANG) 1, ANG2, vascular endothelial growth factor A (VEGF)], which reprogram them to become highly angiogenic and arteriogenic accessory cells (Avraham-Davidi et al., 2013; Hamm et al., 2013). The expression and composition of these vessel- and tissue-derived cytokines are tightly regulated at the ischemic site, and may critically affect the angiogenic and arteriogenic function of macrophages (Folkman, 2006; Saharinen et al., 2010; Saharinen and Alitalo, 2011). For example, ANG1-mediated macrophage reprogramming resulted in the repression of the oxygen-sensitive prolyl hydroxylase domain (PHD) protein 2 (PHD2). PHD2 repression promoted a M2-like, proarteriogenic phenotype of macrophages by activating canonical nuclear factor ‘kappa-light-chain-enhancer’ of activated B-cells (NF-kB) signaling (Takeda et al., 2011). Furthermore, ANG1-mediated *Phd2*-repression enhanced the expression of ANG-receptor TIE2, which amplifies ANG-dependent TIE2 signaling in a positive feedback loop and hence promoted vessel maturation (Hamm et al., 2013). In addition, local tissue hypoxia and endothelial cell derived signals might maintain this regulatory circuit by promoting TIE2 expression (Lewis et al., 2007; He et al., 2012).

In many different models of angiogenesis, M2-like alternatively activated macrophages supported the proliferation and migration of endothelial cells and vessel sprouting (Jetten et al., 2014; Fantin et al., 2010; Marchetti et al., 2011). Further to serving as a source of the potent angiogenic mediators such as VEGF and fibroblast growth factor (FGF)-2 (Schulze-Osthoff et al., 1990; Xiong et al., 1998; Dirkx et al., 2006), IL-4-driven alternatively activated macrophages promoted the release of VEGF from the tissue matrix, thereby enhancing the sprouting of vessels (Zajac et al., 2013). This was shown to be as a result of a blockade of the tissue inhibitor of metalloproteinase 1 (TIMP1) gene expression, that promotes the secretion of highly angiogenic matrix metalloproteinase (MMP) 9, which in turn results in the release of matrix-sequestered angiogenic growth factors such as VEGF and FGF-2 from the tissue (Zajac et al., 2013). Release of macrophage-derived MMP9 might be further augmented by hypoxia, as MMP9 expression is known to be governed by hypoxia inducible factor (HIF) 2a(alpha)-signaling (Yang et al., 2010).

In addition to the above described proangiogenic role of IL-4-dependent M2-like macrophages (Zajac et al., 2013; Jetten et al., 2014), non-IL-4 stimulated macrophages also have important roles. In this context, ANG2-dependent TIE2-signaling in macrophages was also found to promote angiogenesis in models of inflammation and cancer (Coffelt et al., 2010, 2011; Mazzieri et al., 2011; Krausz et al., 2012). Additionally, hypoxia was found to enhance ANG2 expression in murine and human macrophages, which may subsequently boost their proangiogenic function (Fang et al., 2009).

**Figure 1:**
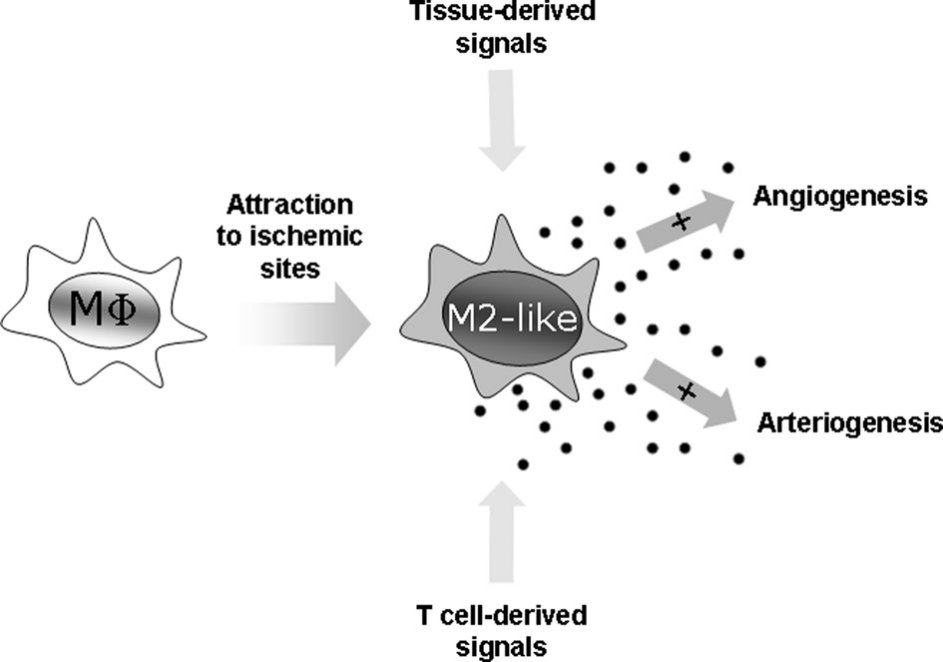
**Macrophages as angiogenic and arteriogenic accessory cells.** Macrophages are attracted to ischemic sites where they are transformed into potent angiogenic and arteriogenic accessory cells by tissue- and T cell-derived signals.

These data demonstrate that macrophage cell function is critically involved in angio- and arteriogenesis (Figure 1). Given the role of T cell-derived cytokines on macrophage polarization and activation it is obvious that alteration in T cell activation will also affect angiogenesis and arteriogenesis, and will thus bring another level of complexity to the effect of immune cells on vascular biology (Starnes et al., 2001; Naldini et al., 2003; Stabile et al., 2003, 2006; Facciabene et al., 2012).

## Macrophages as glucose and lipid sensors

Whilst cytokines and chemokines are the main drivers of the activation and function of macrophages, recent studies have revealed that macrophages also respond to environmental cues in the form of small metabolites such as glucose, lipids, and sodium chloride (to be discussed further in the following section). These small metabolites also influence programming of macrophages into either classical or alternative subsets, and can thus modulate macrophage function.

Macrophages infiltrate and reside in nearly every tissue, including adipose. Accompanied with the observation that macrophages accumulate within adipose tissue with obesity (Weisberg et al., 2003; Xu et al., 2003), there has been great interest on the effect of lipids on macrophage function and activation. Macrophages take up lipids via scavenger receptors, such as CD36 and scavenger receptor A (SR-A). This process is not subject to a negative feedback mechanism and as such, where there is excess lipid present macrophages can become loaded with lipid and form pro-atherogenic foam cells (Nagy et al., 2012). In cases of overnutrition, where the adipose tissue is overwhelmed with nutrients resulting in various amounts of cellular stress (reviewed by Odegaard and Chawla, 2013), macrophages accumulate within adipose tissue and subsequently switch from an alternative activated (M2) phenotype to a classically activated (M1), suggesting that excess fat can enhance the activation of inflammatory signaling pathways (Lumeng et al., 2007a). This has also been demonstrated by *in vitro* experiments, where incubation of macrophage with free fatty acids led to the activation of Toll-like receptor 4 signaling, NF-kB activation and subsequently fatty acid-induced insulin resistance (Shi et al., 2006; Pal et al., 2012). The JNK signaling pathway has additionally been shown to be involved in the activation of inflammatory M1 macrophages and the development of obesity and insulin-resistance (Han et al., 2013). This was also shown by the deletion of JNK1 in hematopoietic-derived cells, which subsequently resulted in protection against diet-induced inflammation and insulin resistance without affecting obesity (Solinas et al., 2007).

Under normal conditions, the transcriptional effect of the uptake of lipids by macrophages is the activation of peroxisome proliferator activator receptors (PPAR) (Ricote et al., 1998). In humans, there are three PPAR subtypes (α, δ, and γ), which are expressed in a variety of cell types and tissues. These three PPAR's act as transcription factors and coordinate the transcription of molecules important for every facet of fatty acid metabolism (reviewed by Desvergne et al., 2006). In terms of macrophage activation, both PPAR-δ and -γ have been shown to be especially important in modulating alternative macrophage activation (reviewed extensively by Nagy et al., 2012). In a recent study, PPAR-δ was implicated in *Salmonella* replication, where upon infection M2 macrophages had elevated PPAR-δ expression. As the deletion of PPAR-δ in macrophages prevented the replication and survival of the bacteria, the authors propose that *Salmonella* have evolved to utilize the metabolic state of M2 macrophages in order to survive (Eisele et al., 2013). PPAR-γ seems to be of particular importance to alternative macrophage activation, as its expression is induced by IL-4 (Huang et al., 1999). It has subsequently been shown that signal transducer and activator of transcription 6 (STAT6), the downstream transcription factor of IL-4 signaling, physically interacts with PPAR-γ at transcriptionally important regions of M2 signature genes to augment their expression (Szanto et al., 2010). Macrophage-specific PPAR-γ knockout mice have also been shown to have an attenuated M2 phenotype: the expression of M2 signature genes such as *Arg1* were blunted, which was coupled with a reduced fatty acid oxidative metabolism (Odegaard et al., 2007). Surprisingly PPAR-γ knockout mice had an increased weight gain on a high fat diet, which was attributed to a decrease in the number of alternatively activated macrophages, and thus a decrease in the homeostatic capacity provided by these macrophages under conditions of overnutrition to ensure that efficient lipid metabolism is occurring (Odegaard et al., 2007). It is tempting to speculate that PPAR-γ-dependent signaling pathways may empower the macrophages to efficiently clear cholesterol from tissue (Martel et al., 2013).

In addition to obesity, the onset of diabetes (type 1 or 2) is closely linked to macrophage activation and function. Type 2 diabetes in particular is closely linked to macrophage function, where the infiltration of macrophages into adipose tissue and their modulation of the inflammation level of this tissue greatly contributes to the development of insulin resistance (Arkan et al., 2005; Kanda et al., 2006; Weisberg et al., 2006; Lumeng et al., 2007b). In addition, macrophages have been shown to directly infiltrate the pancreatic islet and subsequently modulate the function of this organ to promote the onset of diabetes (Ehses et al., 2007). Conversely, glucose itself seems to have an influence on macrophage function (de Souza et al., 2008; Kanter et al., 2012). Furthermore, the state of glucose metabolism within the cell is also important for macrophage polarization. Whilst M1 macrophages rely on glycolytic metabolism *via* HIF1a (Cramer et al., 2003), M2 macrophages utilize fatty acid oxidation via PPAR (as discussed above; Vats et al., 2006). More recently, two studies have identified pathways important for mediating the metabolic activity of macrophages, and thus their activation state. Haschemi et al. demonstrated that the carbohydrate kinase-like (CARKL) protein controls M1-M2 activation by directing the metabolic reprogramming of the macrophage from oxidative phosphorylation to glycolysis (Haschemi et al., 2012). It has also been shown that the mammalian target of rapamycin (mTOR), a master signaling pathway involved in growth and metabolism, is involved in regulating the switch from M2 to M1 activation (Byles et al., 2013). These studies all point toward glucose itself being a “physiological insult” which influences macrophage activation and eventually, their homeostatic function.

## Macrophages act as local sensors and regulators of electrolyte composition in the skin interstitium

Recently, Machnik et al attributed a new role to macrophages in regulating internal body fluids (Machnik et al., 2009, 2010). The conventional key regulator for salt and water regulation is the kidney. This traditional concept is based on the idea that body fluids readily equilibrate and that Na^+^ is the major extracellular cation and holds water in the extracellular space due to its osmotic activity. Consequently, it is believed that renal excretion of excess Na^+^ is sufficient to govern intravascular and interstitial electrolyte content. Thus, researchers interested in the regulation of intravascular and interstitial fluids have previously focused on mechanisms as to how the kidneys handle Na^+^. In contrast to this view, there is now ample evidence that Na^+^ is stored without commensurate water retention in the skin (reviewed in Titze and Machnik, 2010; Titze, 2014). This suggests that the electrolytes in the skin do not readily equilibrate with plasma, and hence escape renal homeostatic control. These findings support the idea that the intravascular and interstitial spaces are two distinct extracellular electrolyte compartments which are regulated separately. In a series of experiments by our laboratory, we have identified that the electrolyte composition of the skin is regulated by macrophages and by its local interstitial lymph capillary system (Machnik et al., 2009, 2010; Wiig et al., 2013).

Macrophages infiltrate to the sites of Na^+^ and Cl^−^ overload in the skin which display a hypertonic microenvironment, indicating that the salt-gradient may be the driving force of macrophage cell attraction (Muller et al., 2013). These recruited macrophages sense the interstitial electrolyte composition and subsequently upregulate the transcription factor, tonicity enhancer binding protein [TonEBP, also termed nuclear factor of activated T-cells 5 (NFAT5)], which is an essential transcription factor required for the expression of osmoprotective genes in response to hypertonicity-induced osmotic stress (Halterman et al., 2012).

The molecular events which lead to NFAT5 activation has been the focus of many studies. The p38/mitogen activated protein kinase (MAPK) signaling pathway is one which is activated by osmotic stress in both mammalian (Han et al., 1994) and yeast cells [Brewster et al. (1993); *via* its MAPK homolog high osmolarity glycerol response protein 1 (HOG1)]. This has been confirmed in many other studies (Shapiro and Dinarello, 1995; Nadkarni et al., 1999; Ko et al., 2002; Morancho et al., 2008; Kuper et al., 2009; Roth et al., 2010; Kleinewietfeld et al., 2013). However, what remains to be shown are the macrophage-specific molecular events which occur upon osmotic stress, upstream of the activation of p38/ MAPK. One possibility is MAPK1 phosphatase-1 (MKP-1), which augments p38/ MAPK signaling upon high concentrations of NaCl (Zhou et al., 2008). Whether this molecule is also important for macrophages has not been shown. In addition to p38/ MAPK, several other signaling cascades have been induced in response to osmotic stress. These include the phosphatidylinositol 3-kinase signaling cascades (Irarrazabal et al., 2004), protein kinase A dependent processes (Ferraris et al., 2002), as well as a Rac1/osmosensing scaffold (Zhou et al., 2011). More recently, a sucrose nonfermenting-1-related serine/ threonine kinase (SIK1) was identified as a sensor of extracellular Na^+^ gradients, subsequently transducing this information into signaling cascades which modulates cellular function (Sjostrom et al., 2007). Importantly, this axis has been demonstrated in macrophages, where inhibition of SIK1 activity was shown to affect M2 regulatory macrophage activation (Clark et al., 2012). Also, serum glucocorticoid kinase-1 (SGK1) is another salt-inducible kinase (reviewed extensively by Lang et al., 2006) which has recently been identified as an important modulator of IL-17 producing CD4^+^ T helper (Th17) cell activation (Wu et al., 2013). How the salt-inducible kinases such as SGK1 and SIK1 interact with NFAT5 in macrophages is unknown. Furthermore, how these signaling cascades are induced and interplay with each other and which effect they have on NFAT5 activation especially in macrophages is yet to be elucidated.

**Figure 2:**
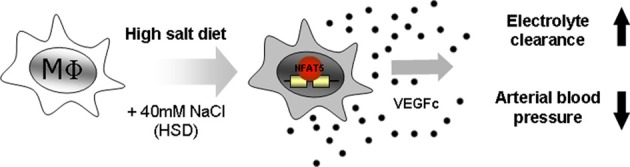
**Macrophages act as local sensors and regulators of electrolyte composition in the skin interstitium.** Macrophages are attracted to tissues with high concentrations of salt. Under these conditions NFAT5 is activated in macrophages. NFAT5 then drives the expression of vascular endothelial growth factor C (VEGFc), resulting in the hyperplasia of skin lymph capillaries. This results in the clearance of electrolytes from the skin and subsequently a reduction in blood pressure.

Our laboratory has demonstrated one biological outcome for the activation of NFAT5 in macrophages. The induction of NFAT5 in macrophages of the skin was shown to directly govern the expression of vascular endothelial growth factor C (VEGF-C), resulting in the hyperplasia of lymph capillaries *via* and interaction with the VEGF receptor 3 (VEGFR3) (Wiig et al., 2013). Similar to a breakdown of the renal salt and water excretion, failure of this local extrarenal macrophage-dependent control mechanism to regulate interstitial electrolyte and water homeostasis, resulted in arterial hypertension and massive disturbances in skin electrolyte composition (Machnik et al., 2009, 2010; Wiig et al., 2013). Thus, these data indicate that the traditional model for electrolyte and water homeostasis, which considers the intravascular and interstitial space as one functional extracellular compartment, is an oversimplification. Instead, the interstitial space is a distinct compartment that relies on tissue-specific regulatory mechanisms for controlling its electrolyte content; this role being fulfilled by macrophages (Figure 2).

Recently, it has become possible to measure the concentration of Na^+^ in the tissue of humans *via* a non-invasive technique using ^23^Na-MRI (Kopp et al., 2012a). These studies revealed that there is an increased local sodium storage in humans that suffer from hyperaldosteronism, hyernatriemia, and hypertension (Kopp et al., 2012a,b, 2013). Harnessing the potential of macrophages to regulate salt and water balance may therefore be of interest to physicians that aim to treat arterial hypertension as well as salt-balance disorders.

## Conclusion

The new homeostatic functions reviewed here, extends the classical role of macrophages as cells which remove foreign microorganisms from the body, to those which tightly regulate the microenvironments of the body to ensure correct blood supply and concentrations of metabolites, electrolytes, and maintain proper tissue function. In light of the amount of overnutrition and excessive dietary intake of sugar, salt and saturated fats in western civilizations, further understanding of the molecular details by which macrophages sense these metabolites is warranted. The identification of other metabolites and electrolytes which influence macrophage polarization and function may reveal new mechanisms by which diseases such as hypertension, type II diabetes and autoimmune diseases may occur.

### Conflict of interest statement

The authors declare that the research was conducted in the absence of any commercial or financial relationships that could be construed as a potential conflict of interest.
